# Safety and Short-Term Efficacy of Irreversible Electroporation and Allogenic Natural Killer Cell Immunotherapy Combination in the Treatment of Patients with Unresectable Primary Liver Cancer

**DOI:** 10.1007/s00270-018-2069-y

**Published:** 2018-08-27

**Authors:** Yumei Yang, Zilin Qin, Duanming Du, Yumin Wu, Shuibo Qiu, Feng Mu, Kengcheng Xu, Jibing Chen

**Affiliations:** 1 Department of Interventional Therapy, The First Affiliated Hospital of Shenzhen University, Shenzhen Second People’s Hospital, No. 3002 of SunGang West Road, FuTian, Shenzhen, 518035 China; 2 Chongqing Health Service Center, Chongqing, 400020 China; 3 Biotherapy Center, Fuda Cancer Hospital of Jinan University, Guangzhou, 510665 China; 4 Department of Oncology, Fuda Cancer Hospital of Jinan University, Guangzhou, China; 5 Fuda Cancer Institute, Guangzhou, China

**Keywords:** Irreversible electroporation, Natural killer cells, Primary liver cancer, Immunotherapy, Clinical trial

## Abstract

**Purpose:**

This study aimed to investigate the safety and short-term efficacy of irreversible electroporation (IRE) combined with allogenic natural killer (NK) cell immunotherapy in the treatment of patients with unresectable primary liver cancer.

**Materials and Methods:**

Between October 2015 and December 2016, 40 patients were enrolled and randomly allocated to either the IRE group (*n* = 22) or the IRE–NK group (*n* = 18). All adverse events experienced by the patients were recorded; the changes in tumor biomarkers [AFP, CA 19-9, circulating tumor cells (CTCs)], lymphocyte number and function, quality of life, clinical response, progression-free survival (PFS) and overall survival (OS) were assessed.

**Results:**

Patients who received combination therapy exhibited significantly longer median PFS and OS than who just received IRE (PFS 15.1 vs. 10.6 months, *P* < 0.05, OS 17.9 vs. 23.2 months, *P* < 0.05). The combination therapy of IRE and NK cell immunotherapy significantly reduced CTCs and increased immune function and Karnofsky performance status.

**Conclusion:**

Our data suggest a novel, promising combination therapy using IRE and allogenic NK cell immunotherapy. Larger clinical trials are required to confirm these conclusions.

**Electronic supplementary material:**

The online version of this article (10.1007/s00270-018-2069-y) contains supplementary material, which is available to authorized users.

## Introduction

Primary liver cancer (PLC) is the second leading cause of cancer deaths in less developed countries and is the sixth leading cause of cancer deaths among men in developed countries. Its incidence has been increasing every year, with China alone accounting for about 50% of the total estimated new liver cancer cases [1, 2]. Surgery, although regarded as the gold standard treatment, is suitable for less than 20% of patients with PLC due to multiple tumors, metastasis, hepatic function compromise, or other deleterious factors [3]. Most PLCs are unresectable when diagnosed. Transcatheter arterial chemoembolization (TACE) and sorafenib treatment have shown promise in randomized controlled trials in selected unresectable PLC populations [4–6]. However, TACE-related toxicity has limited its use to intermediate stage PLC [7], and sorafenib extends the overall survival (OS) by only 3 months. Subsequent efforts in drug developments have failed [8]. Percutaneous thermal ablation is considered the optimal treatment choice for focal unresectable PLC of early stage; however, there is a risk of collateral thermal damage to sensitive adjacent organs: when the target lesion is adjacent to vessel, the heat-sink effect can cause the ablation of the lesion to be incomplete, the shape and size of the ablation zone may be unpredictable [9]. This challenging clinical scenario warrants new, safe, effective, and life-prolonging strategies for patients with PLCs.

In recent years, irreversible electroporation (IRE), a new, nonthermal and minimally invasive technique developed on the basis of reversible electroporation technology, has been used increasingly in the clinic, which is not affected by the heat-sink or cold-sink effect that may lead to incomplete ablation especially in perivascular tumor cells, and causing little damage to normal tissues, such as the gall bladder, bile duct [10–12]. Therefore, IRE permits the treatment of tumors unsuitable for surgical resection or thermal therapies. Moreover, the large number of tumor-specific antigen remaining in situ after IRE lacks thermal denaturation, resulting in a more potent immune response than thermal ablation which enhances the therapeutic outcome. Many studies have shown immunocyte infiltration in ablated areas following IRE treatment [13–15]. Studies in rats have suggested that IRE treatment changes the status of cellular immunity with a significant increase in peripheral lymphocytes and serum cytokines [16]. But the recurrence of PLCs following IRE remains a common phenomenon in the clinical setting [17].

The clinical application of natural killer (NK) cell-based immunotherapy is in its initial stage, with allogenic NK cells increasingly pursued for adoptive cellular therapies since the discovery of the renowned killer immunoglobulin-like receptor (KIR)/histocompatibility antigens (HLA) mismatch [18]. Our previous research demonstrated the safety and efficacy of NK cell-based immunotherapy in the treatment of PLC patients [19]. IRE combined with allogenic NK cell immunotherapy has been applied to the treatment of pancreatic cancer and has proved to be safe and efficacious [20]. However, the combination therapy has not been applied to the treatment of PLC. NK cells are enriched in human livers, forming 30–50% of the intrahepatic lymphocytes. The immune surveillance exerted by NK cells is crucial to the immune functions and defense of the liver against cancer [21]. On this basis, we surmise that the IRE with allogenic NK cell immunotherapy may be a promising combination therapy for PLC.

This is the first prospective study which assessed the safety and short-term efficacy of IRE and allogenic NK cell immunotherapy combination in the treatment of patients with unresectable PLCs.

## Materials and Methods

### Patients

**Fig. 1:**
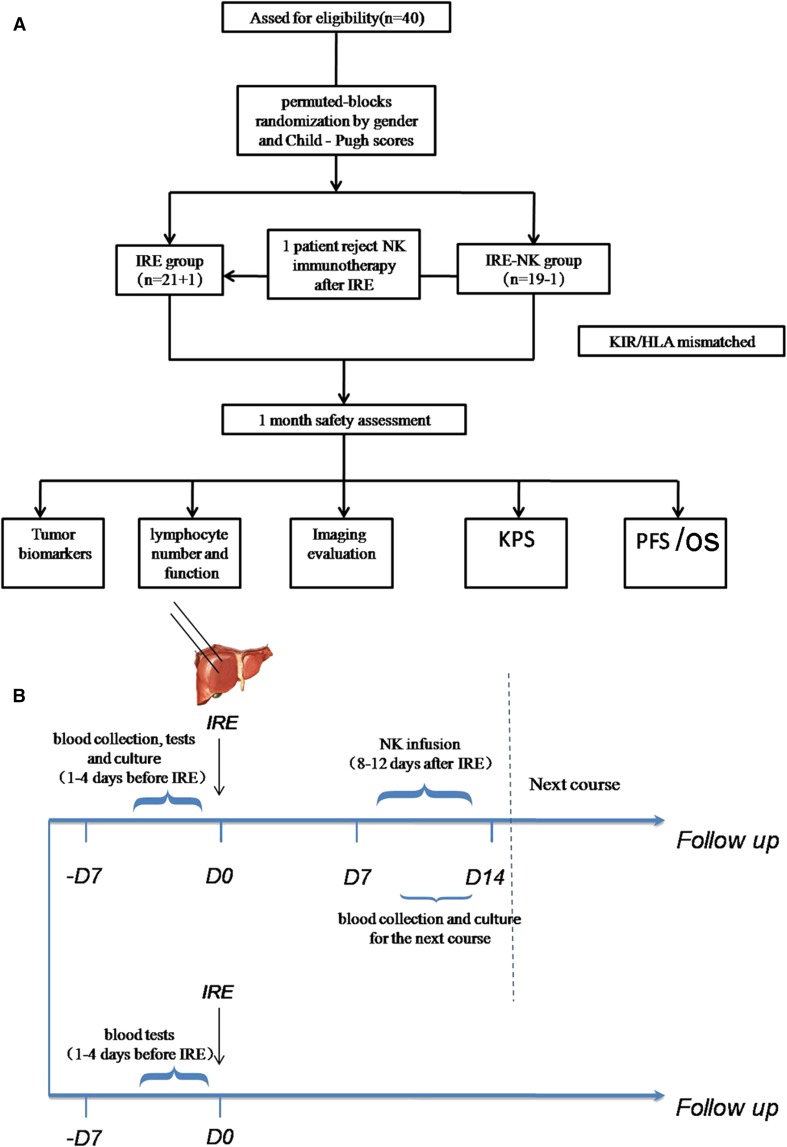
**A** Study design flowchart. A total of 40 patients were included and stratified by center. Patients were allocated to groups by a permuted blocks randomization protocol. One patient allocated to the IRE–NK group rejected NK immunotherapy following IRE since failing to find out a KIR ligand donor and was thus excluded from the IRE–NK group (*n* = 18) and included in IRE group (*n* = 22). **B** Treatment schedule. Peripheral blood was collected to obtain the NK cells 1–4 days prior to IRE treatment. NK cell immunotherapy was started within 14 days following blood collection and given for 3 consecutive days (8–12 days after IRE). The next course of collection started 1 day before the last infusion of the previous course

This prospective study enrolled 40 patients with PLC between October 2015 and December 2016. The inclusion criteria were as follows: (1) 20–80 years old; (2) clear diagnosis of PLC based on imaging and pathological findings with tumor lesion < 10 cm; (3) unresectable PLC, which was defined as the impossibility of completely removing the tumor or retaining a sufficient liver remnant to maintain liver function; (4) liver function classified as Child–Pugh class A or B; (5) not more than 3 intrahepatic lesions or 3 extrahepatic metastatic lesions, no invasion of the portal vein, the hepatic vein trunk or secondary branches, and expected survival > 6 months; and (6) Karnofsky performance status (KPS) > 60. Exclusion criteria: (1) serious abnormalities in liver, lung, heart or kidney function; (2) massive ascites or brain metastasis; (3) acute or chronic infection; (4) patients who had blood coagulation disorders, severe anemia, or other primary tumors and (5) patients who were positive for HIV, HTLV-1, syphilis, tuberculosis, or parasitic blood infections. The primary endpoint of the current study was progression-free survival (PFS) and OS of the treated lesions, as there have been no studies of direct comparison of IRE and IRE–NK regarding PFS or OS in the treatment of patients with unresectable PLC in a randomized clinical trial format. In our institution, approximately 75 patients with PLC were treated by IRE annually. We assumed that among those patients, 38 patients could be enrolled in this study. And assuming the drop rate as 5%, size of the target population was determined as 40. The patients were allocated to groups by stratified permuted blocks randomization method on gender and Child–Pugh scores (Child–Pugh A and Child–Pugh A) (Fig. 1A). The enrolled patients were allocated to either the IRE or the IRE–NK group. This study was approved by the Institutional Review Board of Fuda Cancer Hospital and complies with the provisions of the Declaration of Helsinki. All patients provided written informed consent. This trial was registered at ClinicalTrials.gov (Trial No. NCT03008343).

### Irreversible Electroporation Procedure

All patients underwent muscle relaxants and general anesthesia. IRE was performed using an IRE ablation system (NanoKnife™ system, model HVP01; AngioDynamics, Queensbury, New York, USA). The main configuration includes a high-voltage current generator (maximum power output of 3 kV, 50 A), an electrocardiogram (ECG) synchronization (an AccuSyne^R^ synchronizer Device, AccuSync Medical Research Corporation, Milford, Connecticut, USA), a 15-cm pulse start probe (Model 20400103) and a 15-cm pulse standard probe (Model 20400104). All the percutaneous ablations were guided by computed tomography (CT SOMATOM Definition 64 AS; Siemens Medical Solutions, Forchheim, Germany) combined with ultrasound (US; IU22; Philips Medical Systems, Bothell). The number of probes, the method of inserting the needle, and the operation parameters were determined by the pre-operation plan of IRE. The distance between the electrodes was 1.5–2.5 cm [22], and the effective exposure probe distance was 1.5–2.5 cm. The parameters of the IRE generator were set as follows: the pulse length was 70–90 μs, the pulse repetition was 70–90, and the average electric field intensity was 800–2200 v/cm. One or more pullbacks were performed if the target region was > 2 cm in diameter. All pulses were delivered during the ventricular refractory period to avoid the occurrence of arrhythmias. Following the ablation, CT imaging was performed to confirm the ablation range was more than 0.5–1 cm on the edge of the tumor; the patient was then transferred to the intensive care unit for 24 h, and then transferred to the general ward if their vital signs were stable. Relevant treatment was administered if there were any complications. Two surgeons with 4–8 years of experience in image-guided tumor ablation performed all procedures.

### IRE–NK Therapy Procedure

The relatives of the enrolled patients were informed, and their peripheral blood was collected from KIR/HLA-mismatched donor to obtain the NK cells 1–4 days prior to IRE treatment [21]. The human high activity NK cell in vitro preparation kit (Hank Bioengineering Co. Ltd, Shenzhen, China) was used to expand and activate NK cells from peripheral blood mononuclear cells in vitro to prepare NK cells of higher quantity, purity, and activation, namely highly activated NK (HANK) cells (Electronic supplementary material [ESM] Fig. 1). NK cell immunotherapy was started within 14 days following blood collection and given for 3 consecutive days (8–12 days following IRE). The next course of collection started 1 day before the last infusion of the pervious course. The 18 patients in the IRE–NK group received continuous treatment of 4 courses of NK cell immunotherapy (Fig. 1B).

### Adverse Events

Complications during treatment and post-treatment were evaluated in accordance with the Common Terminology Criteria of Adverse Events, v4.0.

### Tumor Biomarkers

Circulating tumor cells (CTCs), serum α-fetoprotein (AFP) and CA 19-9 were detected at 1–4 days pre-treatment and at 1 month post-treatment. AFP normal range: 0–5.8 IU/ml, CA 19-9 normal range: 0–39 U/ml. CTCs was defined as CD45-negative, CK-positive, and CD326-positive cells and was quantified by flow cytometry (FACSCanto™ II; BD, Grand Island, NY, USA).

### Imaging

Dynamic CT/magnetic resonance imaging (MRI) abdominal scans were obtained prior to IRE after 1 month and then at 3 months post-treatment. The modified response evaluation criteria in solid tumors (mRECIST) was used to assess the tumors. To accurately observe the therapeutic effects, the total area of all tumors before and after treatment was compared. According to the criteria, clinical effects were divided into complete response (CR), partial response (PR), stable disease (SD), and progressive disease (PD). PFS was calculated from the first treatment to the time at which patients were found to meet the criteria for PD. CR + PR represent the effective rate (RR). The results were evaluated independently by 2 experienced radiologists, who were blinded to all other patient history. In the case of disagreement, they collectively read the film and reached a consensus.

### Karnofsky Performance Status

KPS evaluation was recorded before and after treatment as an index of quality of life (QOL).

### Detection of Immune Function

One to four days prior to IRE treatment, and 1 month following the final NK cell transfusion, peripheral blood (2 ml) was collected and assessed using flow cytometry (FACSCanto™ II; BD, Grand Island, NY, USA). The tested indices included lymphocyte number and function in the patients’ peripheral blood. A BD Multitest 6-color TBNK reagent (No. 644611) was used to detect the number of CD3 + CD4 + cells, CD3 + CD8 + cells, total CD3 + cells, CD3 − CD19 + cells, and CD3 − CD16 + CD56 + cells. The BD cytometric bead array (CBA) human Th1/Th2 cytokine kit II (No. 551809) was used to detect the expression levels of interleukin-2 (IL-2), IL-4, IL-6, IL-10, tumor necrosis factor (TNF), and interferon-*γ* (IFN-*γ*). The tests were performed according to the protocols in the instruction manuals. Results above or within the reference range were defined as normal; results below the reference range were defined as immune dysfunction. For the IRE group, peripheral blood was drawn 1–4 days prior to IRE and 1 month following IRE; blood was drawn from the IRE–NK group 1–4 days prior to IRE and 1 month following NK cell therapy.

### Statistical Analysis

The primary end point of the study was PFS and OS; PFS was calculated from the date protocol treatment was started until the date of death or of the first evidence of radiographic disease progression. OS was calculated from the date the treatment was started until the date of death. Differences were considered significant at *P* < 0.05. The basic characteristics, adverse events, and RR of the two groups were compared using the Chi-square test; immunity detection, tumor biomarkers, and KPS data were presented as the mean ± standard deviation and compared using the Student’s *t*-test. SPSS version 13.0 (IBM; Armonk, NY, USA) was used for the statistical analyses, and measurement data were expressed as the mean ± standard deviation. GraphPad Prism 5 (GraphPad Software, San Diego, CA) was used to plot graphs and analyze the PFS and OS rate.

## Results

### Patient Characteristics

A total of 40 patients were included and randomly assigned to the IRE group (*n* = 21) or the IRE–NK group (*n* = 19). One patient allocated to the IRE–NK group rejected NK immunotherapy following IRE since failing to find out a KIR ligand donor and was excluded from IRE–NK group (*n* = 18). The patient was then included in the IRE group (*n* = 22). Patient demographics were not statistically different between the two groups (Table 1).

**Table 1:** Patient characteristics

Factor	IRE (*n* = 22)	IRE–NK (*n* = 18)	*P* value
Gender			*P *= 0.676
Male	12	11	
Female	10	7	
Median age (years)	54	57	*P *= 0.723
Child–Pugh stratification			*P *= 0.822
Class A	9	8	
Class B	13	10	
Clinical stage (AJCC)			*P *= 0.737
III	8	5	
IV	14	13	
Histology			*P *= 0.750
HCC	13	9	
ICC	9	9	
Tumor number			*P *= 0.286
Solitary	8	3	
Multifocal	14	15	
Maximal diameter largest tumor (cm) Mean ± SD	4.73 ± 1.62	4.81 ± 1.61	*P *= 0.871
Maximal diameter largest tumor (cm)			*P *= 0.523
< 5	14	9	
5–10	8	9	
AFP (IU/ml)			*P *= 0.6389
< 200	6	6	
200–400	2	3	
≥ 400	14	9	
KPS score			*P *= 0.714
70	12	8	
80	7	8	
90	3	2	
TACE	15	14	*P *= 0.499

*AJCC* American joint committee on cancer staging system, *AFP* α-fetoprotein, *KPS* Karnofsky performance status, *TACE* transarterial chemoembolization

### Progression-Free Survival and Overall Survival

**Fig. 2:**
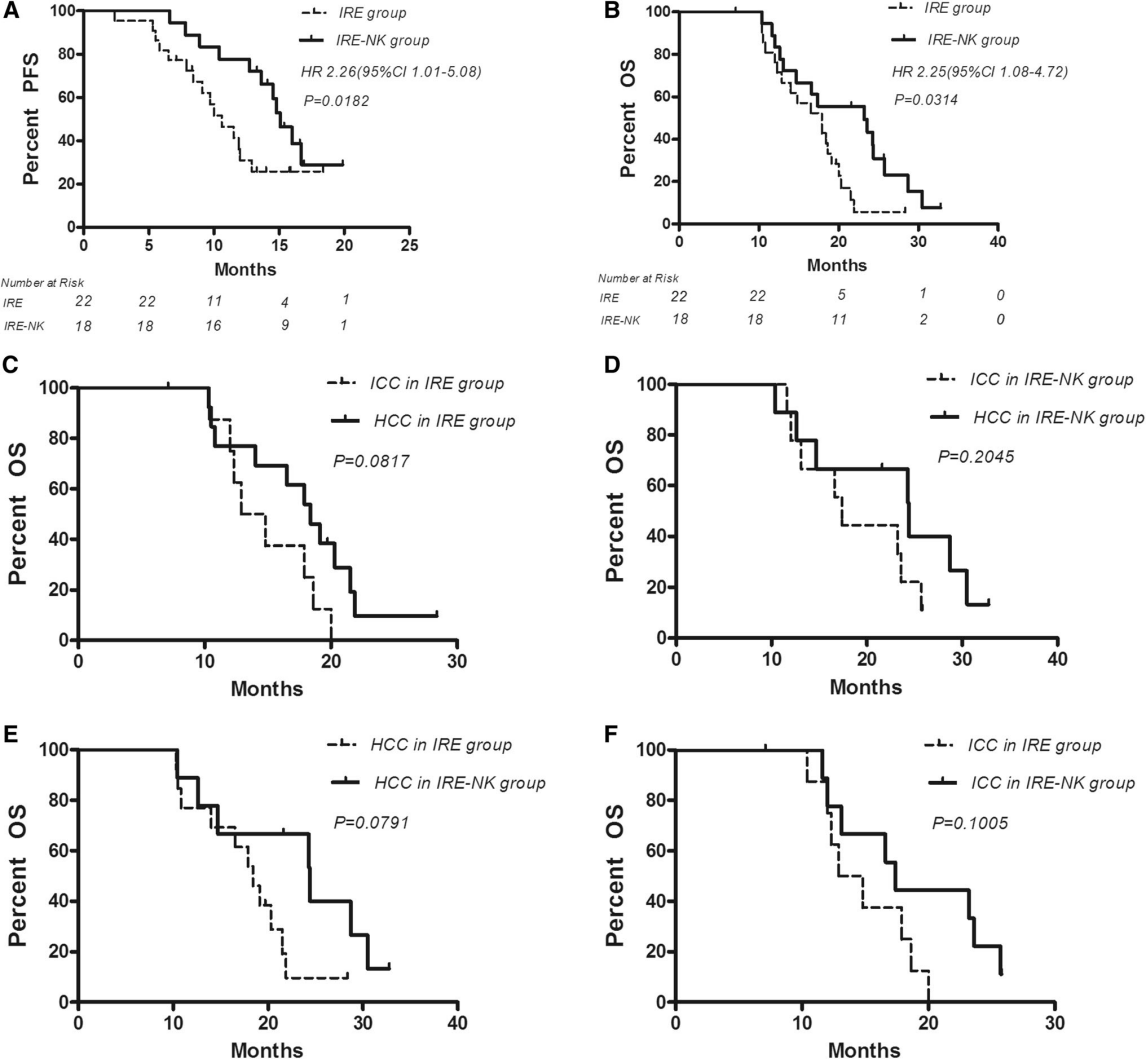
**A** Progression-free survival shows that PFS in the IRE–NK group was significantly higher than in that of IRE group during follow-up period (*P* < 0.05). The median PFS in the IRE group was 10.6 and 15.1 months in the IRE–NK group. **B** Median OS for IRE group and IRE–NK group was 17.9 versus 23.2 months, with HR 2.25 (95% CI 1.08–4.72). **C** OS of HCC and ICC in IRE group was not significantly different. **D** OS of HCC and ICC in IRE–NK group was not significantly different. **E** OS of HCC between IRE group and IRE–NK group was not significantly different. **F** OS of HCC between IRE group and IRE–NK group was not significantly different. *OS* overall survival, *PFS* progression-free survival, *HR* hazard ratio

The median PFS of patients who underwent IRE was 10.6 months, which is shorter than that of patients who underwent IRE combined with allogenic NK cell immunotherapy (15.1 months). The PFS was significantly different between these two groups (*P* = 0.018). One-year OS for IRE group and IRE–NK group was 66.7 and 77.8%, respectively, with median OS 17.9 versus 23.2 (*P* = 0.031). However, OS of HCC or ICC in the interior group and inter-group was not significantly different (Fig. 2).

### Response to Treatment

Treatment response was evaluated according to the mRECIST guidelines by 2 experienced radiologists. The inter-observer reproducibility between readers 1 and 2 was almost in perfect agreement (*κ* = 0.858). The intra-observer reproducibility based on reader 1’s twice was almost in perfect agreement (*κ* = 0.903). Therefore, all outcomes were based on the measurements taken by the first radiologist. After follow-ups for 3 months, 1 patient in the IRE group and 3 patients in IRE–NK group displayed CR; PR was recorded in 14 patients of the IRE group and 13 patients in the IRE–NK group. Development of new lesions was observed in 1 patient from the IRE group at 2.4 months post-treatment using a contrast-enhanced CT scan. The RR in the IRE–NK group (88.9%) was higher than in the IRE group (68.2%) (*P* = 0.15). The representative results are shown in Table 2.

**Table 2:** Clinical response 3 months post-treatment

Group	Total	CR	PR	SD	PD	RR (%)
IRE group	22	1	14	6	1	68.2
IRE–NK group	18	3	13	2	0	88.9

*CR* complete response, *PR* partial response, *SD* stable disease, *PD* progressive disease, *RR* response rate

### Comparison of Immune Function

**Fig. 3:**
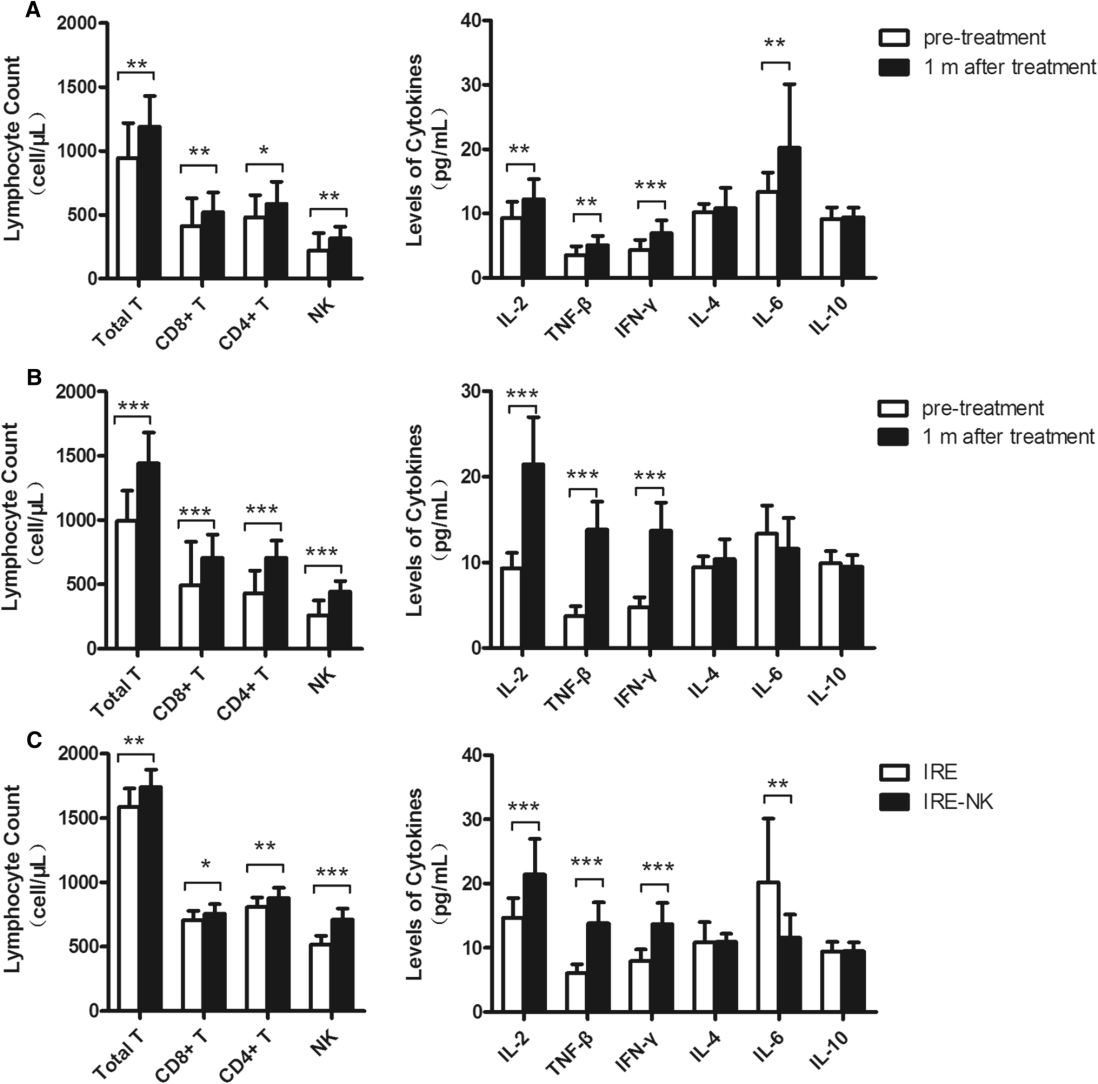
**A** Changes in total T cells, NK cells, Th1 cytokines, and IL-6 were significant in the IRE group 1 month post-treatment; **B** Changes in total T cells, NK cells, and Th1 cytokines were significant in the IRE–NK group 1 month post-treatment; **C** At 1 month post-treatment, total T cells, NK cells, and Th1 cytokines were significantly higher, while IL-6 was lower in the IRE–NK group compared to the IRE group. *P < 0.05, **P < 0.001, ***P < 0.0001

In IRE group, the absolute number of lymphocyte subsets, Th1 cytokine (IL-2, IFN-*β*, IFN-*γ*) levels and IL-6 1 month post-treatment were higher than that before treatment. A similar and more obvious variation tendency was observed in IRE–NK group except IL-6. No significant changes in IL-4 or IL-10 were observed in both groups (Electronic supplementary material [ESM] Table 1).There were no significant differences in the lymphocyte count or Th1 cytokine levels between the two groups pre-treatment (*P* > 0.05),but which were higher in the IRE–NK group compared to the IRE group post-treatment (*P* < 0.05,) (Figure 3).

### Safety Evaluation

The adverse events measured post-treatment include pain, pleural effusion, ascites, fatigue, and fever. The IRE group had 6, 1, 3, 3, and 10 patients with these symptoms, respectively. In the IRE–NK group, 4, 4, 4, 5, and 11 patients, respectively, exhibited these symptoms (ESM Fig. 2). Groups were compared using the Chi-square test; there was no difference among the two groups (*P* = 0.8707). The reaction degree was grade 1 or 2 and was all relieved after symptomatic treatment. All adverse reactions mainly occurred within 2 weeks following IRE, and only a few patients had fever following NK cell immunotherapy.

### Changes in Karnofsky Performance Status

**Fig. 4:**
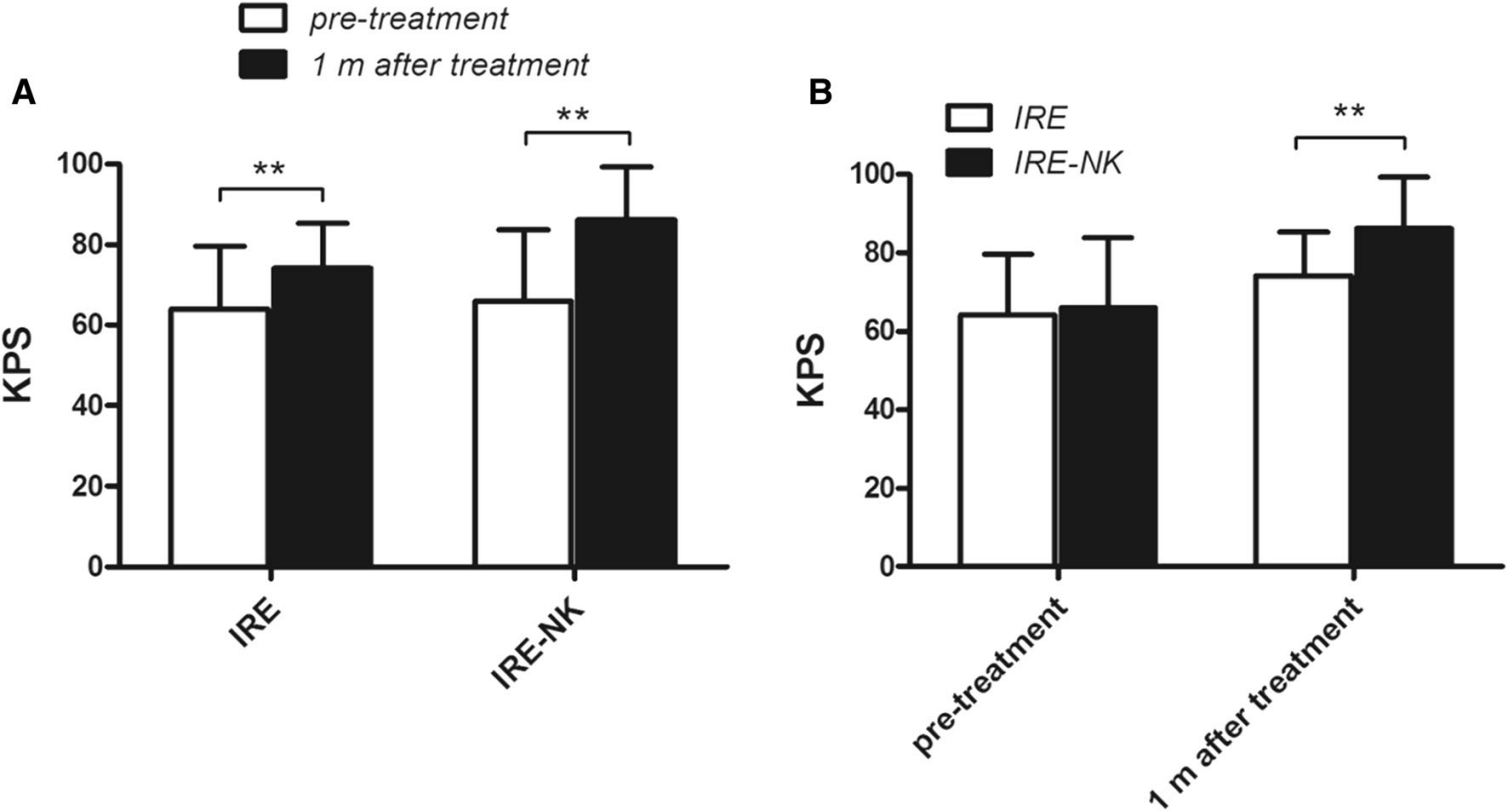
**A** Changes in KPS in the IRE and IRE–NK groups. KPS was significantly increased in both groups 1 month post-treatment. **B** KPS was significantly increased in the IRE–NK group compared to the IRE group 1 month post-treatment. **P < 0.01

The pre-treatment KPS of the IRE group and IRE–NK group was 64.14 ± 15.6 and 65.96 ± 17.8 (*P* > 0.05), respectively. The KPS was 74.13 ± 11.2 and 82.21 ± 13.2, respectively, 1 month post-treatment. The KPS of both groups was improved significantly post-treatment compared to pre-treatment. The KPS was significantly higher in the IRE–NK group at 1 month post-treatment (Fig. 4A, B).

### Changes in Tumor Biomarkers

**Fig. 5:**
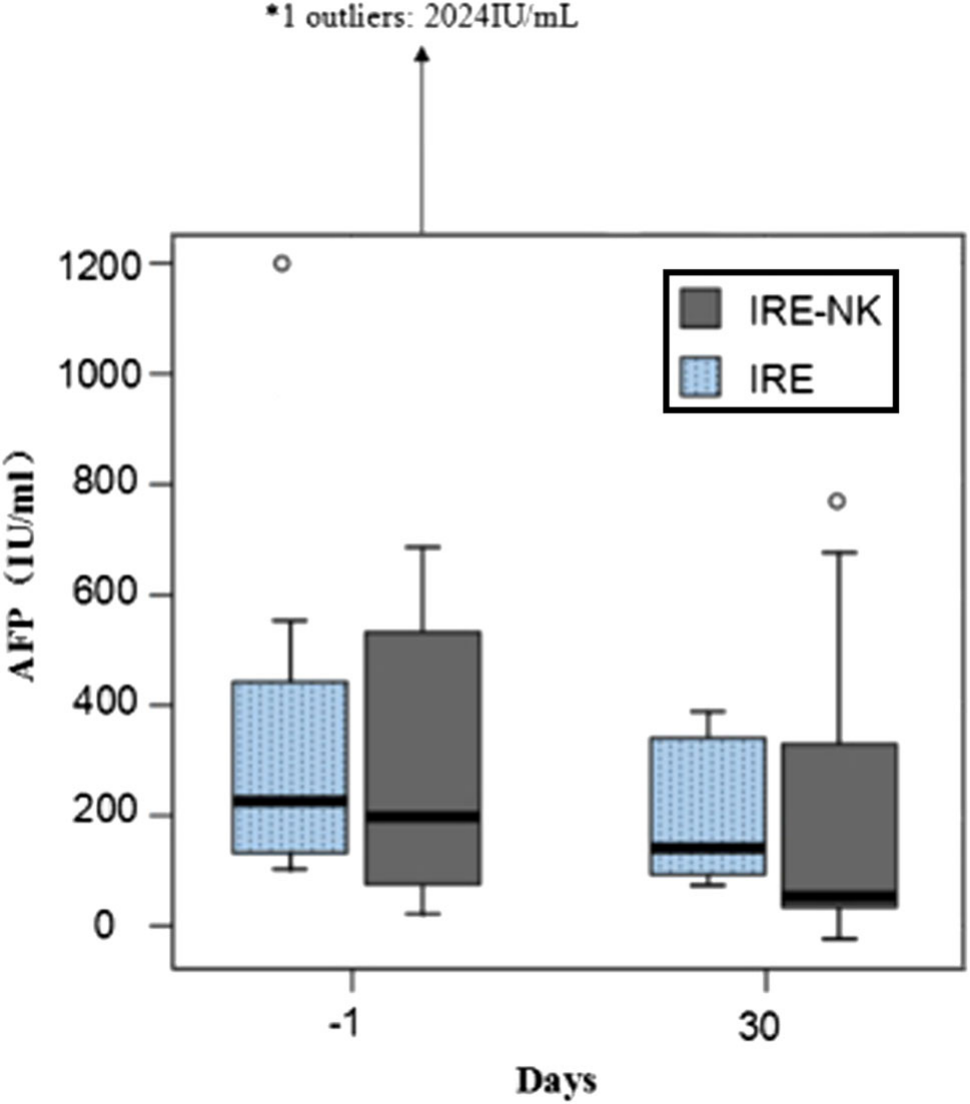
Box-and-whisker plot shows AFP value before and after treatment. AFP was significantly decreased in both groups (*P* < 0.05)

**Fig. 6:**
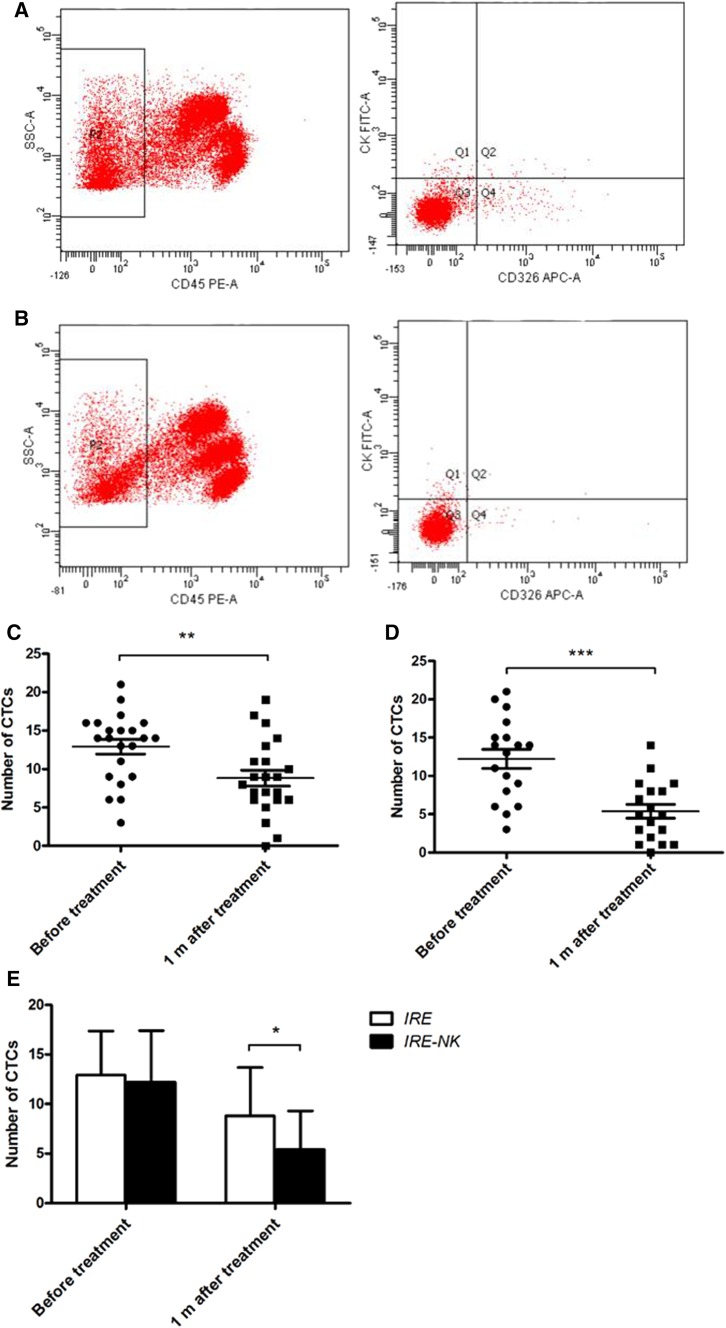
**A** Proportion analysis of CTCs prior to treatment; **B** Proportion analysis of CTCs 1 month post-treatment; the number of CTCs was decreased significantly in the IRE (**C**) and IRE–NK groups (**D**) 1 month post-treatment and is lower in the IRE–NK group (**E**). *P < 0.05, **P < 0.001, ***P < 0.0001

AFP (Fig. 5) and CTCs expression 1 month following treatment were lower in both groups (*P* < 0.05). There was no difference in biomarkers between the two groups prior to treatment (*P* > 0.05). However, 1 month post-treatment, CTCs (Fig. 6) expression was significantly lower in the IRE–NK (*P* < 0.05). No significant changes in CA 19-9 were observed in either group in this study.

## Discussion

As a nonthermal ablation and minimally invasive therapy, IRE has brought therapeutic promise in unresectable PLC. Its high complete ablation rate and long recurrence-free period not affected by blood flow absorption makes it a powerful clinical tool. However, there were still a number of recurring patients in prior studies. [23–25] Immunosuppression in patients with PLC is an important factor leading to poor prognosis, recurrence, and metastasis. [26, 27] Murine studies have suggested that IRE therapy has a greater therapeutic effect in immunocompetent patients compared to those lacking a complete immune system [28]. Immunotherapy represents a promising new approach in the treatment of cancers [29]. However, immunotherapy alone cannot effectively prevent the progression of tumor, and it is difficult to counteract large tumor load. In recent years, multiple studies confirmed that the combination of local ablation treatments with immune stimulation appears to be a robust approach since immunotherapy can effectively target tumor cells of blood circulation that cannot be controlled by local ablation [21, 30].

As the first line of defense against tumor in human body, NK cells have a good prospect in the field of immunotherapy because they do not need the stimulation of specific antigens. NK cells can recognize major histocompability complex class I (MHC-I) molecules on the cell surface by KIR, and the activation of NK is inhibited when the two molecules are combined. Therefore, NK cells cannot be activated unless the MHC antigen on the surface of tumor cells mutates or disappears. It is generally accepted that KIR/HLA-mismatched NK cells are more effective in the treatment of malignant tumors and do not provoke graft-versus-host response. Our previous research demonstrated that KIR-mismatched HANK cell immunotherapy is a safe therapy which improves the immune function of patients with liver cancer and reduces the rate of tumor metastasis and recurrence [19].

The pathophysiology of IRE is still not completely understood. In theory, the specific tumor cell antigen will not degrade from hyperthermia following the nonthermal IRE ablation; it may be well preserved as whole tumor cell vaccine. IRE ablation of tumor tissue retains the extracellular matrix, including vascular structure and lymphangion. Inactivated vascular endothelial cells can regenerate and revascularize following IRE, providing a structural basis for a variety of infiltrating immune cells. [13, 31, 32] Innate immune effector cells carry antigens from lymphatic vessels to lymph nodes, and then, activating anti-tumor T cell immune response in the draining lymph nodes, killer T cells can return to tumor site with circulation to eradicate residual tumor cells and prevent distant metastasis. Compared with the thermal ablation technique, IRE more effectively activates the immune function and induces a tumor immune response. In our study, the increase in Th1 cytokines and total T cells in the IRE group demonstrates that IRE treatment increases tumor susceptibility to host immunity. Furthermore, the increase in Th1 cytokines and total T cells in the combined therapy group was significantly higher than in the IRE treatment alone. The Th1 cytokines increased in this study, indicating that IRE combined with NK cell immunotherapy may shift the balance of Th1/Th2 and activate cellular immunity. No changes in Th2 cytokines were measured in the IRE–NK group; however, IL-6 in IRE group was significantly higher than prior to treatment. We speculate that it may be related to the immune activation mediated by damage-associated molecular pattern (DAMP). Mitochondrial DNA, high mobility group box-1(HMGB1), heat shock protein, and s100 proteins can be released by DAMP mode from necrotic cells. Then stimulate the corresponding TLR to produce cytokines including IL-6. Bulvik et al. [33] reported that peak IL-6 levels of IRE were three times higher than RF 6 h after liver ablation of female C57BL/6 mice. Their study suggests that IRE not only protects large blood vessels, but also preserves microcirculation and enhances the ability of cytokines included IL-6 to enter the systemic circulation. As we know that IL-6 has potential tumorigenic ability, positively correlated with the progression of liver cancer, and negatively regulates the body’s immunological function. In this study, No significant increase in IL-6 was observed in IRE–NK group, indicating that NK cell immunotherapy could regulate and improve the cellular immune response, positively regulate the immune system function and improve the anti-tumor effect. However, the specific mechanism of its role needs further study.

In this study, the response rate in 3 months reached 88.9 and 68.2% for IRE–NK and IRE, respectively. PFS and OS were significantly improved in the IRE–NK group, demonstrating the synergistic effect of these two therapies. Combined therapy seems superior to single IRE therapy in short-term efficacy. However, OS of HCC and ICC seems to have no significant differences (*P* > 0.05) in the interior group or inter-group. Considering that this study is a small sample of preliminary exploratory clinical study, the conclusions may be accidental and only for reference and need to be confirmed by larger clinical trials. In our previous study, the number of NK treatments also impacted on the efficacy of the therapy; the PFS rate was significantly higher in patients who received more than 4 courses of NK cell therapy compared to those who received less than four [19]. Thus, the patients enrolled in this study were treated with 4 courses of NK cell therapy. However, the optimal number of treatment courses and the duration of treatment remain to be optimized.

The combination of NK cell immunotherapy immediately after IRE in the treatment of patients with unresectable PLC has not been reported. Its safety must be observed and verified since the immune status and development background of patients with PLC are complex. In this report, the side effects observed were not significant between both groups. The reaction degree was grade 1 or 2, and all symptoms were relieved following symptomatic treatment. The adverse reactions mainly occurred within 2 weeks following IRE treatment, and only a few patients had fever following NK cell immunotherapy. This suggests that IRE combined with allogenic NK cell immunotherapy is well tolerated.

Biomarkers have an important role in the diagnosis, predicting the prognosis, and monitoring of the patients with PLC. AFP is one of the most common biomarkers for hepatocellular carcinoma (HCC). In our study, serum AFP significantly decreased in both groups and is lower in the NK-IRE group compared to the IRE group 1 month post-treatment. This indicates a better prognosis of the combination therapy. In addition, the CTC level is a biomarker that shedding from the primary tumor site and entering the peripheral blood circulation which positively correlated to tumor size [34]. We used CD326 as the positive selection while CD45 as the negative selection in the detection of peripheral blood CTCs to reduce the incidence of false negative. The decrease in CTCs observed in the present study may therefore reflect the improved efficacy of combination therapy. CA 19-9 is always used to monitor patients with ICC. However, there was no significant change in CA 19-9 in the present study. It appears that CA 19-9 is not sensitive to the outcome or prognosis of these treatments. However, the number of patients is too little, and some of the patients were already undergoing other therapies, such as surgery and TACE, before they were recruited. A larger sample of patients is needed to reach a more definitive conclusion.

In conclusion, this single-center prospective study has demonstrated the short-term safety and efficacy of IRE combined with allogenic NK cell immunotherapy for unresectable PLCs. However, the study has the following limitations: a small sample size and a short follow-up time. Therefore, long-term efficacy should be measured by extending the group of patients and increasing the period of follow-ups.

## Electronic supplementary material

Below is the link to the electronic supplementary material.

Supplementary material 1 (DOCX 6176 kb)
