# Supplementary material for: Safety and Short-Term Efficacy of Irreversible Electroporation and Allogenic Natural Killer Cell Immunotherapy Combination in the Treatment of Patients with Unresectable Primary Liver Cancer
Source: Cardiovasc Intervent Radiol. 2018 Aug 27;42(1):48–59. doi: 10.1007/s00270-018-2069-y (PMC6267679; doi:10.1007/s00270-018-2069-y)
Supplement: Supplementary file 1 — Supplementary material 1 (DOCX 6176 kb) [file 270_2018_2069_MOESM1_ESM.docx]

Electronic Supplementary Material

**Appendix A1: ESM** **Figure1.****Flow chart of** **NK cells culture in vitro**

80 mL peripheral blood was drawn from allogenic donors

600g centrifugation for 15 minutes, with plasma in the upper layer and peripheral blood mononuclear cells(PBMCs) in the middle layer

**Centrifugation**

The upper plasma was inactivated in water bath at 56 ℃ for 30 minutes, then mixed with plasma treatment solution (HK-001), then placed at room temperature for 1-2 hours and 400g centrifugation for 10 minutes. Which was used to configure HANK cell culture medium.

**Inactivation**

2 portions were cultured in parallel, each T75 culture bottle was added 4-5×10^7^PBMC, 40 ml cell - culture medium and 1 NK synergist (HK A3)

**Day 1**

Replaced culture bottle and culture medium on Day 3, so that the cell concentration was about 1.0 × 10^6^

**Day 3**

Replaced culture bottle and culture medium on Day 3, so that the cell concentration was about 1.0 × 10^6^.The first sterility test was performed on Day 6 and an HK-A3 was added on Day 7 . Supplementary culture medium on Day 9 and Day 12

**Day5-12**

Cell counting and quality control inspection were performed on Day 12 of culture, and the quality indicators include ≥8 billion total cells with ≥90% living cells, ≥80% CD3-/CD56+ cells (detected by flow cytometry), ≤1 EU/ml endotoxin, ≥80% cell killing activity against K562 target cells, and absence of bacteria, fungi, or mycoplasma in cell culture

The NK cells were divided into three groups and intravenously infused into the patients on three consecutive days on Day13-15 ( 8–12 days following IRE)

**Infusion**

**Appendix A2: ESM table1. Comparison of** **lymphocyte number and function**

| Lymphocyte test  items | IRE group | | IRE-NK group | |
| --- | --- | --- | --- | --- |
|  | pre-treatment | post-treatment | pre-treatment | post-treatment |
| Number (cell/μL): |  |  |  |  |
| Total T cells | 945.9±273.7 | 1186.9±243.0** | 993.4±235.3 | 1443.1±237.9*** |
| CD8+T cells | 410.4±218.5 | 519.6±153.8** | 492.3±338.5 | 707.7±179.8*** |
| CD4+T cells | 480.8±172.9 | 585.5±174.2* | 430.9±175.9 | 706.8±134.1*** |
| NK cells | 221.0±136.6 | 315.7±90.1** | 260.1±115.5 | 442.2±85.7*** |
| Function (pg/mL): |  |  |  |  |
| IL-2 | 9.3±2.5 | 12.4±3.0** | 9.3±1.8 | 21.5±5.5*** |
| TNF-β | 3.6±1.3 | 5.1±1.5** | 3.7±1.2 | 13.8±3.3*** |
| IFN-γ | 4.3±1.6 | 6.9±2.0*** | 4.8±1.2 | 13.7±3.3*** |
| IL-6 | 13.3±3.1 | 20.2±9.9** | 13.4±3.3 | 11.6±3.6 |
| IL-4 | 10.2±1.3 | 10.9±3.2 | 9.5±1.3 | 10.4±2.4 |
| IL-10 | 9.2±1.8 | 9.5±1.5 | 9.9±1.5 | 9.5±1.4 |

NK cell, natural killer cell; IL, interleukin; TNF, tumor necrosisfactor; IFN, interferon.

* P < 0.05. ** P < 0.01. *** P < 0.001.

**Appendix A3: Reader Agreement in the Assessment of Response Evaluation**

The results of tumors response were evaluated independently by 2 experienced radiologists(readers 1 and 2, with 15 and 13 years of experience in CT/MRI abdominal interpretation, respectively), who were blinded to all other patient history. The agreement level between the two readers was measured by using k coefficient. k values for level of agreement was defined as follows: 0.81-0.99, almost perfect agreement; 0.61-0.80, substantial agreement; 0.41-0.60, moderate agreement; 0.21-0.40, fair agreement; and 0.01-0.20, slight agreement. Meanwhile, to assess the intra-observer reproducibility, reader 1 repeated the film twice during a 2-week period.

The inter-observer reproducibility between readers 1 and 2 was almost perfect agreement (Kappa=0.858). The intra-observer reproducibility based on reader 1’s twice was almost perfect agreement (Kappa=0.903).Therefore, all outcomes were based on the measurements made by the first radiologist.

The detailed data are as follows：

|  |  | Reader 2 | | | | Total |
| --- | --- | --- | --- | --- | --- | --- |
|  |  | CR | PR | SD | PD |  |
| Reader 1 | CR | 4 | 0 | 0 | 0 | 4 |
|  | PR | 0 | 25 | 2 | 0 | 27 |
|  | SD | 0 | 0 | 7 | 1 | 8 |
|  | PD | 0 | 0 | 0 | 1 | 1 |
| Total |  | 4 | 25 | 9 | 2 | 40 |

|  |  | The Second Diagnosis of Reader 1 | | | | Total |
| --- | --- | --- | --- | --- | --- | --- |
|  |  | CR | PR | SD | PD |  |
| First Diagnosis of  Reader 1 | CR | 4 | 0 | 0 | 0 | 4 |
|  | PR | 0 | 25 | 2 | 0 | 27 |
|  | SD | 0 | 0 | 7 | 1 | 8 |
|  | PD | 0 | 0 | 0 | 1 | 1 |
| Total |  | 4 | 25 | 9 | 2 | 40 |

**Appendix A4: ESM Figure1.** **Adverse events**


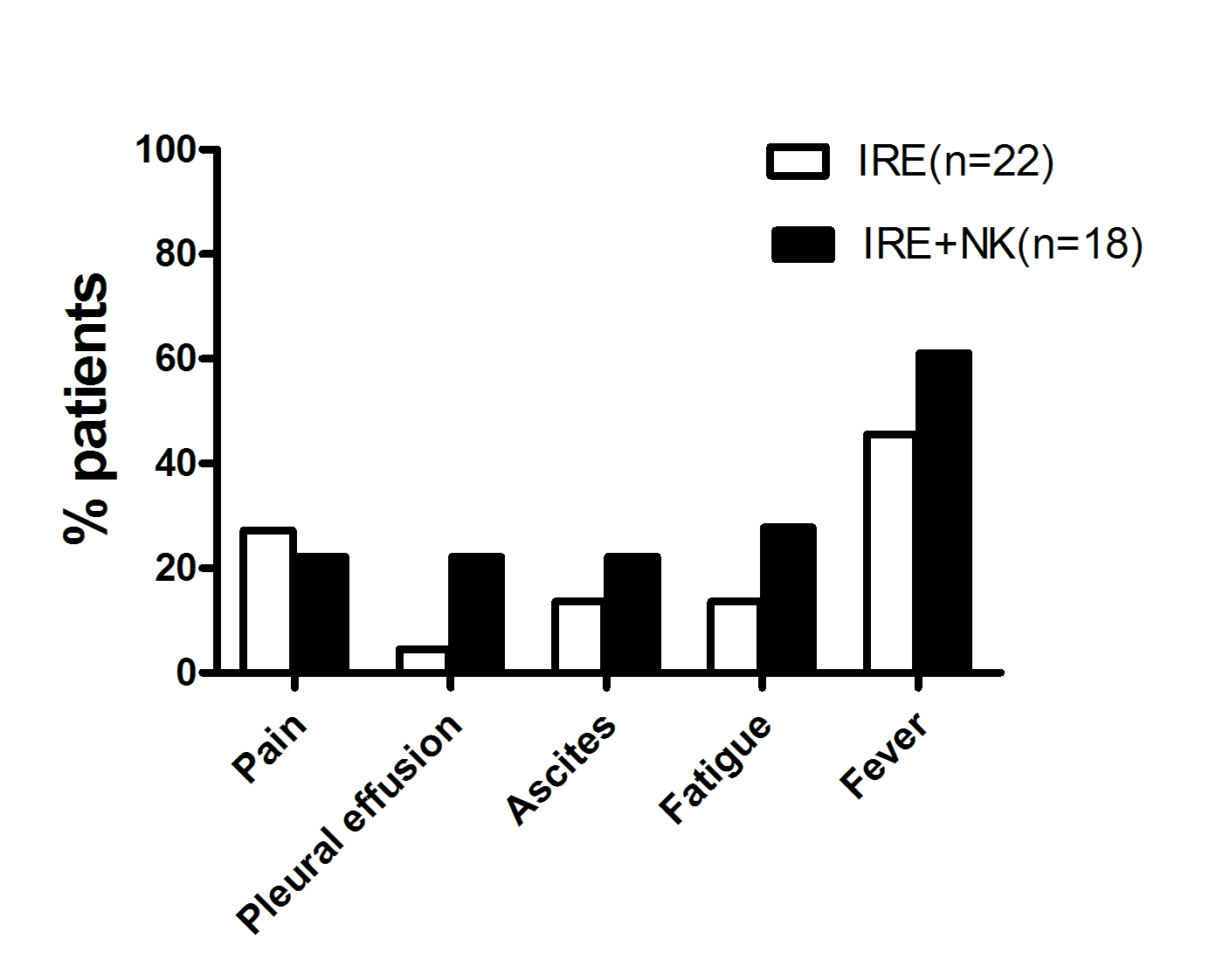


Adverse events between IRE group and IRE-NK group were compared using the chi-square test; there was no difference among the two groups (P= 0.8707)
